# Evidence for a Trade-Off Strategy in Stone Oak (*Lithocarpus*) Seeds between Physical and Chemical Defense Highlights Fiber as an Important Antifeedant

**DOI:** 10.1371/journal.pone.0032890

**Published:** 2012-03-22

**Authors:** Xi Chen, Charles H. Cannon, Nancy Lou Conklin-Brittan

**Affiliations:** 1 Department of Biological Sciences, Texas Tech University, Lubbock, Texas, United States of America; 2 Key Lab in Tropical Ecology, Xishuangbanna Tropical Botanic Garden, Chinese Academy of Sciences, Yunnan, People's Republic of China; 3 Department of Human Evolutionary Biology, Peabody Museum, Harvard University, Cambridge, Massachusetts, United States of America; New York State Museum, United States of America

## Abstract

Trees in the beech or oak family (Fagaceae) have a mutualistic relationship with scatter-hoarding rodents. Rodents obtain nutrients and energy by consuming seeds, while providing seed dispersal for the tree by allowing some cached seeds to germinate. Seed predation and caching behavior of rodents is primarily affected by seed size, mechanical protection, macronutrient content, and chemical antifeedants. To enhance seed dispersal, trees must optimize trade-offs in investment between macronutrients and antifeedants. Here, we examine this important chemical balance in the seeds of tropical stone oak species with two substantially different fruit morphologies. These two distinct fruit morphologies in *Lithocarpus* differ in the degree of mechanical protection of the seed. For ‘acorn’ fruit, a thin exocarp forms a shell around the seed while for ‘enclosed receptacle’ (ER) fruit, the seed is embedded in a woody receptacle. We compared the chemical composition of numerous macronutrient and antifeedant in seeds from several *Lithocarpus* species, focusing on two pairs of sympatric species with different fruit morphologies. We found that macronutrients, particularly total non-structural carbohydrate, was more concentrated in seeds of ER fruits while antifeedants, primarily fibers, were more concentrated in seeds of acorn fruits. The trade-off in these two major chemical components was more evident between the two sympatric lowland species than between two highland species. Surprisingly, no significant difference in overall tannin concentrations in the seeds was observed between the two fruit morphologies. Instead, the major trade-off between macronutrients and antifeedants involved indigestible fibers. Future studies of this complex mutualism should carefully consider the role of indigestible fibers in the foraging behavior of scatter-hoarding rodents.

## Introduction

The seed represents a critical stage in the life cycle of a plant as they allow the embryo to migrate before germination. Because the mother tree typically invests considerable resources into the endosperm and embryo, seeds are often rich in nutrients. A wide range of animals consumes tree seeds to gain these nutrients, which could potentially kill the embryo [1]–[3]. Among these animals, scatter-hoarding rodents have a unique co-evolutionary relationship with many tree species. They consume tree seeds, while also playing a dual role as seed-dispersers. Fagaceae, including beech trees and oaks, produce dry nuts rich in nutrients and have a mutualistic relationship with scatter-hoarding rodents [4]–[6]. Rodents forage for the fallen nuts and either eat the seeds immediately or bury them in caches for later consumption. Seed dispersal is then accomplished when the seed germinates before being consumed. A complex suite of fruit and seed characteristics, particularly the phytochemical composition of the seed, directly affects the foraging behavior of rodents [7]. Previous research has demonstrated that seed size, the concentration of chemical antifeedants like tannin, and overall macronutrient content play important roles in whether the seed is consumed in situ or whether and how far it is removed and [8]–[9].

Macronutrients, including protein, lipid and carbohydrate, are important investments for seed germination and early plant establishment, meanwhile seed predator foraging strategies are affected and influenced by them [1]. A higher concentration of macronutrients typically leads to higher predation rates and dispersal distances [5], [7], [10]. Antifeedants, on the other hand, represent an equally important chemical investment to prevent the seeds from being consumed but without making the seeds completely unpalatable [6]. Tannin, fiber, monoterpenoid and terpenoid are the major phytochemicals used as plant antifeedants [11]. In previous studies, tannins were considered crucial to seeds' chemical defense, reducing seed consumption and inhibiting predators' ingestion [4], [12], [13]. Compare to tannins, the role of fiber as an antifeedant has not been closely examined.

**Figure 1:**
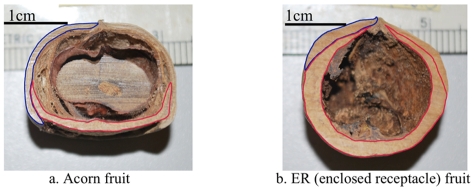
Acorn and Enclosed Receptacle (ER) fruit types. The bar indicates the scale of the seeds. Blue and red contour lines represent the exocarp and receptacle tissue, respectively. a) Acorn fruit represented by *L. calolepis*. The seed develops above the flat receptacle, greatly expanding the exocarp. b) ER fruit represented by *L. xylocarpus*. The seed remains embedded in the developing and greatly expanding receptacle while the vestigial exocarp forms a small apical layer.

**Figure 2:**
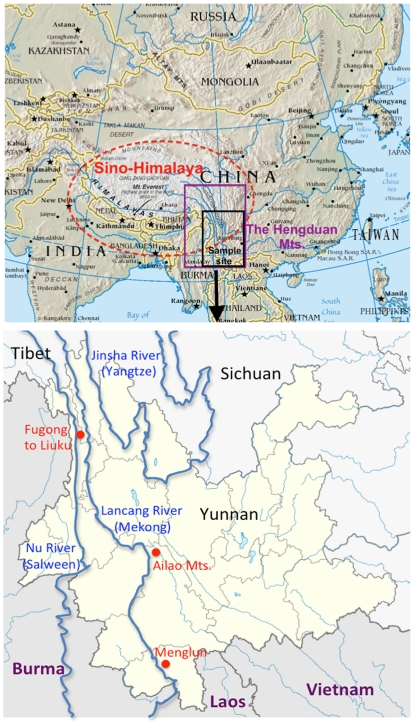
The sample sites in the Hengduan Mts., Sino-Himalaya region. The top figure illustrates the Sino-Himalaya region and the sample sites within the Hengduan Mts. region. The lower figure indicates the three sample sites in Yunnan province.

**Figure 3:**
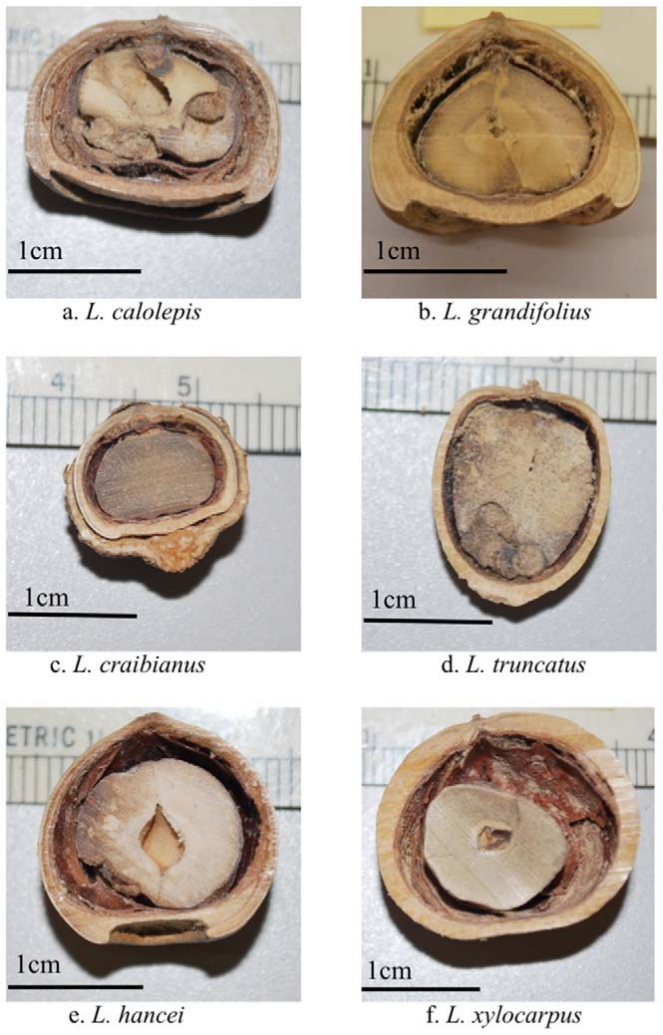
Radial sections of fruit from the six *Lithocarpus* species. Four species had acorn fruits (a, b, c, and e) while two species had enclosed receptacle (ER) fruits (d and f). Collection sites for each species were: Hengduan Mountain region (a–b); the lowland site – Wushigonglichu, Menglun (c–d); the highland site – Ailao Mountains, Jingdong (e–f). The 1cm bar indicates the scale of each fruit.

Two distinct fruit types (Fig. 1) are found within the tropical stone oaks (*Lithocarpus*): acorns and enclosed-receptacle (ER) fruits [14]. ‘Acorns’ are almost identical to temperate oak (*Quercus*) fruits, with the seed enclosed mainly by a thin exocarp or fruit wall, with the receptacle tissue sealing off the base of the fruit. In an ER fruit, the woody receptacle encloses the seed, providing greater mechanical protection. This novel morphology has probably evolved more than one time [15]. Here, we examined whether the investment in seed macronutrients and antifeedants (see Table 1 for list) in these two different types of fruits represents a trade-off between mechanical and chemical protection. Specifically, whether the increased mechanical protection, i.e. species with the ER fruit type, is associated with a decrease in chemical protection, i.e. antifeedants and an increased investment in macronutrients compared to the phytochemistry of seeds from acorn fruits. We directly compared these patterns between two pairs of sympatric species with differing morphologies, one in the lowland and one in the highland forests, along with several other species collected from the Hengduan Mts. region in Yunnan province, southwest China (Figs. 2 and 3).

**Table 1:** Abbreviations of measured macronutrient and antifeedant fractions.

Phytochemical type	Fractions	Abbreviation
Macronutrients	crude protein	CP
	free simple sugar	FSS
	Lipid	Lipid
	total non-structural carbohydrate	TNC
Antifeedants	neutral detergent fiber	NDF
	acid detergent fiber	ADF
	Hemicellulose	HC
	Lignin	Ln
	Cellulose	Ce
	condensed tannin	CT
	bioactive tannin	BT

## Results

### Seed Size of Six Acorn and ER Species

**Figure 4:**
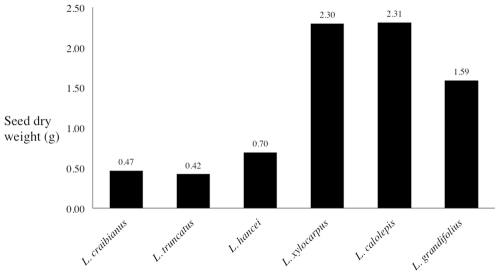
The seed size (dry weight) of six *Lithocarpus* species. *L. craibianus* (acorn) and *L. truncatus* (ER) are sympatric lowland species while *L. hancei* and *L. xylocarpus* are sympatric and co-dominant species in the highlands. *L. calolepis* was also collected in the Ailao Mountains but it is not generally sympatric with the previous two species. *L. grandifolius* was collected on the roadside from Fugong to Liuku.

The seeds of the lowland acorn species, *L. craibianus,* were slightly larger than the sympatric ER species, *L. truncatus* (Fig. 4), while the seeds of the highland ER species, *L. xylocarpus,* were three times larger than the sympatric acorn species, *L. hancei*. The acorn species, *L. calolepis*, which was not sympatric with *L. hancei* and *L. xylocarpus*, has the largest seed size in the analysis.

### Seed phytochemical composition among species

Condensed tannin differed substantially among six species (p[*L. hancei* vs. *L. craibianus*] <0.01, p[*L. craibianus* vs. *L. truncatus*] <0.05, p[*L. hancei* vs. *L. truncatus*] <0.01 and p[*L. truncatus* vs. *L. xylocarpus*] <0.01), while the mean values of other antifeedants were not significantly different (Table 1 and 2). A considerable amount of within-species variance in these values was observed, particularly in the values of condensed tannins in general and particularly for *L. hancei* (see Table 2), which had some of the lowest and the highest levels of condensed tannin. On the other hand, bioactive tannins were relatively consistent within and among species. Among macronutrients, the observed values for most elements were not significantly different among species, except for crude protein and free simple sugar. Both *L. hancei* and *L. truncatus* had greater values for crude protein compared to *L. craibianus* (both p<0.05), while for free simple sugars, *L. hancei* seeds had higher concentrations than *L. craibianus* and *L. xylocarpus* (both p<0.05).

**Table 2:** Seed antifeedant and macronutrient levels for all sampled individuals in six *Lithocarpus* species, expressed at a percentage of field dry weight.

Species Name	Sample	NDF	ADF	HC	Ln	Ce	CT	BT	CP	FSS	Lipid	TNC	Ash
*L. calolepis*	1	24.2	5.8	18.3	3.1	2.7	4.3	9.0	4.7	22.3	0.9	68.2	2.1
*L. craibianus*	1	25.5	2.9	22.6	1.0	1.8	3.0	20.1	3.6	18.1	0.6	68.3	1.9
	2	30.9	3.0	27.9	1.2	1.8	3.0	16.6	4.3	12.3	0.9	62.0	2.0
	3	21.6	3.2	18.4	1.7	1.5	2.3	18.7	4.2	10.7	0.2	71.8	2.2
	4	25.4	3.3	22.1	1.7	1.6	2.7	18.0	3.3	13.4	0.2	68.8	2.2
	5	28.5	3.5	25.0	1.5	2.1	2.2	20.8	4.0	13.1	0.7	64.5	2.3
	6	26.6	2.9	23.8	1.5	1.3	0.8	18.7	3.8	11.9	0.4	66.7	2.4
*L. grandifolius*	1	22.1	2.6	19.5	1.0	1.6	4.4	9.0	7.3	17.6	0.9	67.1	2.6
*L. hancei*	1	25.6	5.0	20.6	2.6	2.4	8.8	8.3	5.9	11.8	0.8	65.6	2.1
	2	26.7	3.8	22.9	1.5	2.3	9	10.4	10.1	24.8	1.2	59.3	2.6
	3	27.3	3.5	23.8	1.4	2.0	9.8	10.4	7.8	13.6	0.8	61.3	2.9
	4	30.2	3.5	26.6	1.5	2.1	10.7	10.4	8.4	26	1.0	58.2	2.2
	5	21.8	2.2	19.5	0.9	1.4	8.4	9.7	7.4	27.9	1.5	67.5	1.8
	6	28.0	4.8	23.1	2.4	2.4	1.3	7.6	9.2	18.4	1.1	60.1	1.7
	7	25.9	2.5	23.4	0.7	1.8	1.4	8.3	8.2	27.7	1.2	63.2	1.5
*L. truncatus*	1	13.0	1.5	11.5	0.3	1.2	0.3	11.1	3.7	18.4	0.5	81.2	1.6
	2	17.8	1.9	15.8	0.3	1.6	0.2	16.6	5.9	17.2	0.7	73.9	1.7
	3	14.2	1.5	12.7	0.3	1.2	0.4	14.6	5.9	20.1	1.0	7.1	1.8
*L. xylocarpus*	1	14.5	2.5	12.0	1.1	1.4	6.8	13.9	4.7	16.5	0.9	78.2	1.6
	2	20.6	2.9	17.7	1.1	1.7	5.0	14.6	3.7	15.7	1.1	73.3	1.3
	3	14.9	1.9	13.1	0.3	1.6	7.5	11.8	3.6	15.3	1.2	78.9	1.4
	4	24.6	2.1	22.4	0.9	1.2	1.8	16.6	3.8	19.9	1.7	68.9	1.0
	5	14.4	2.1	12.3	0.7	1.4	7.1	13.9	4.1	22.0	1.2	79.1	1.1
	6	19.9	3.0	16.9	0.8	2.2	7.3	14.6	4.7	19.2	1.1	73.2	1.1
	7	21.2	2.3	19.0	0.5	1.7	5.3	13.9	5.7	18.8	1.2	70.6	1.3

*All numbers represent as composite measurements of several seeds. Please refer to the material and methods for the calculation of the fractions.*

### Seed phytochemical composition in acorn and ER fruits

Among six species, the two producing ER seeds contained a significantly higher level of total nonstructural carbohydrate than acorn seeds (p<0.001, Table 3 and 4), while neither condensed tannin nor bioactive tannin levels were significantly different between seeds of the two fruit types (both p>0.05). However, the levels of all fiber elements (lignin, hemicellulose and cellulose) and fiber groups, i.e., neutral-detergent fiber (including lignin, cellulose and hemicellulose) and acid-detergent fiber (including cellulose and lignin), were significantly higher in acorn seeds compared to ER seeds (p<0.001, p<0.001, p<0.05, p<0.001, and p<0.001, respectively).

**Table 3:** Mean values of seed antifeedants and macronutrients in six *Lithocarpus* species.

Fruit type	Species Name	NDF	ADF	HC	Ln	Ce	CT	BT	CP	FSS	Lipid	TNC	Ash	Dry weight (g)
Acorn	*L. calolepis*	24.2	5.8	18.3	3.1	2.7	4.3	9.0	4.7	22.3	0.9	68.2	2.1	2.3
	*L. craibianus*	26.4	3.1	23.3	1.4	1.7	2.3	18.8	3.9	13.3	0.5	67.0	2.2	0.5
	*L. grandifolius*	22.1	2.6	19.5	1.0	1.6	4.4	9.0	7.3	17.6	0.9	67.1	2.6	1.6
	*L. hancei*	26.5	3.6	22.9	1.6	2.1	7.1	9.3	8.1	21.5	1.1	62.2	2.1	0.7
ER	*L. truncatus*	15.0	1.7	13.3	0.3	1.3	0.3	14.1	5.1	18.6	0.7	77.4	1.7	0.4
	*L. xylocarpus*	18.6	2.4	16.2	0.8	1.6	5.8	14.2	4.3	18.2	1.2	74.6	1.3	2.3

*All numbers represent as composite measurements of several seeds. Please refer to the material and methods for the calculation of the fractions.*

**Table 4:** ANOVA of seed macronutrients and antifeedants comparing *Lithocarpus* species with different fruit types (acorn vs. enclosed receptacle).

Chemical Components		df	Mean Sq	F value	p-value
Macronutrients	CP	1	14.7	4.1	>0.05
	Lipid	1	0.3	2.7	>0.05
	FSS	1	0.7	0.0	>0.05
	TNC	1	674.2	41.8	<0.001***
Antifeedants	CT	1	2.4	0.4	>0.05
	BT	1	7.2	0.4	>0.05
	NDF	1	434.5	40.5	<0.001***
	ADF	1	10.6	15.0	<0.001***
	Ln	1	5.4	17.4	<0.001***
	HC	1	307.6	30.5	<0.001***
	Ce	1	1.0	6.8	<0.05*

Those values that are designated with a * exhibit a statistical difference at p<0.05, ** for p<0.01, and *** for p<0.001.

### Seed phytochemical composition between sympatric species

The concentration of indigestible fiber, including hemicellulose, lignin, neutral detergent fiber, and acid detergent fiber, was significantly greater in acorn seeds than in ER seeds in both sympatric comparisons (p<0.01, p<0.001, p<0.01 and p<0.001, Table 5), which follows our prediction given that fiber is an effective antifeedant. On the other hand, the concentration of other antifeedants did not follow our predications in every location. The seeds of *L. xylocarpus* (ER fruit type-highland) contained a higher concentration of bioactive tannin compared to seeds of *L. hancei* (acorn fruit type) (p<0.01), while *L. hancei* seeds exhibited significantly higher crude protein level compared to *L. xylocarpus* seeds (p<0.001), a situation opposite to our predictions. However, we observed a significantly higher level of total nonstructural carbohydrate in the seeds of both ER fruits from highland and lowland (*L. xylocarpus* and *L. truncatus*, p< 0.001 and p<0.05). In addition, the concentration of free simple sugar was also higher in *L. truncatus* (ER fruit type) seeds compared to *L. craibianus* (acorn fruit type) (p<0.01). Among antifeedants, condensed tannin, lignin, hemicellulose, as well as neutral and acid detergent fiber were all significant higher in the lowland species *L. craibianus* (acorn fruit type) seeds (p<0.01, p<0.001, p<0.01, p<0.01 and p<0.001 respectively). Between two sympatric highland species, all fiber elements and groups (cellulose, hemicellulose, lignin, neutral detergent fiber and acid detergent fiber) were significantly higher in the seeds of *L. hancei* (acorn fruit type) (p<0.05, p<0.01, p<0.05, p<0.01 and p<0.05 respectively).

**Table 5:** Pair-wise comparisons of seed macronutrients and antifeedants between sympatric *Lithocarpus* species in highland and lowland areas.

		Highland species				Lowland species			
Chemical Components		*L. hancei*	*L. xylocarpus*	df	p-value	*L. craibianus*	*L. truncatus*	df	p-value
Macronutrients	CP	8.1	4.3	9.5	<0.001***	3.9	5.2	2.2	>0.05
	Lipid	1.1	1.2	12.0	>0.05	0.5	0.7	4.6	>0.05
	FSS	21.5	18.2	7.5	>0.05	13.3	18.6	6.3	<0.01**
	TNC	62.2	74.6	11.6	<0.001***	67.0	77.4	3.9	<0.05*
Antifeedants	CT	7.1	5.8	8.9	>0.05	2.3	0.3	5.3	<0.01**
	BT	9.3	14.2	10.2	<0.01**	18.8	14.1	2.6	>0.05
	NDF	26.5	18.6	10.2	<0.01**	26.4	15.0	5.1	<0.01**
	ADF	3.6	2.4	7.9	<0.05*	3.1	1.6	4.3	<0.001***
	HC	22.8	16.2	9.7	<0.01**	23.3	13.3	5.8	<0.01**
	Ln	1.6	0.8	8.1	<0.05*	1.4	0.3	5.0	<0.001***
	Ce	2.1	1.6	11.8	<0.05*	1.7	1.3	4.9	>0.05

L. hancei (acorn fruit type) and L. xylocarpus (ER fruit type) are co-dominant and sympatric species from the highland. L. craibianus (acorn fruit type) and L. truncatus (ER fruit type) are sympatric species from the lowland. CP (crude protein). Those values that are designated with a * exhibit a statistical difference at p<0.05, ** for p<0.01, and *** for p<0.001. Those p values that are bold and italic exhibit an opposite result from prediction.

### MANOVA and Discriminant Function Analysis

**Figure 5:**
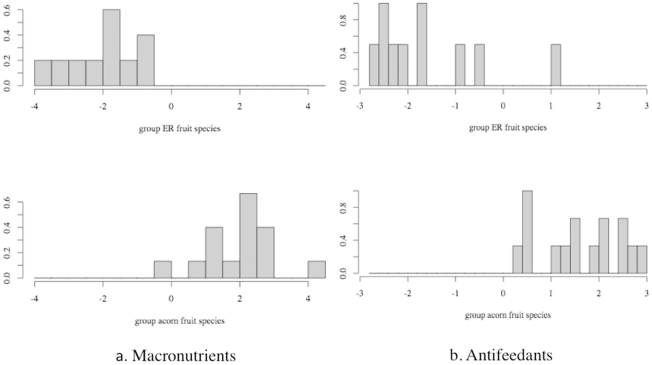
Linear discriminant function analysis of macronutrients and antifeedants between seeds of acorn and ER fruit. Acorn species include *L. hancei*, *L. calolepis*, *L. craibianus* and *L. grandifolius*; ER species are *L. truncatus* and *L. xylocarpus*. See Table 1 for a list of the macronutrients and antifeedants included in this analysis.

Among all six species, even with a small portion of overlapping, acorn and ER fruits were distinctively different in their overall partitioning of antifeedants and macronutrients, with F[_4_], [20] = 20.7, p<0.001 and _F_ _[5]_,[19] = 10.1, p<0.001 respectively (Table 6 and Fig. 5).

When all of the macronutrients are considered together, especially starch and non-starch polysaccharides (total nonstructural carbohydrate minus free simple sugar), seeds from ER fruits had higher concentrations (LD1 = –1.1 and –2.9 respectively, Table 6), representing a greater overall investment. Additionally, where fraction by fraction comparisons between seeds from acorn and ER fruits revealed little importance for lipids (Table 4 and 5), lipid played an important factor interacting with other fractions and contributed to a higher level of macronutrients in ER seeds (LD1 = –1.2).

Among all the antifeedants, hemicelluloses and lignin were two macronutrient fractions responsible for a higher antifeedant level in acorn fruits' seeds (LD1 = 1.4 and 1.0 respectively). Given all antifeedants together, three other fractions (condensed tannin, hydrolysable tannin, and cellulose) contributed to a higher level of antifeedant in ER fruits' seeds, which is in agreement with the previous result (Table 4 and 5). These differences It also explain the large divergence of antifeedant composition between seeds of acorn and ER fruits (Figure 4).

**Table 6:** Linear discriminant function analysis (DA) of macronutrients and antifeedants between seeds of acorn and ER fruits.

Chemical elements		LD1
Macronutrient elements	CP	–0.3
	Lipid	–1.2
	FSS	–1.1
	TNC – FSS	–2.9
Antifeedant elements	CT	–0.3
	BT – CT	–0.5
	HC	1.4
	Ln	1.0
	Ce	–0.4

*Macronutrient elements include crude protein (CP), lipid (Lipid), free simple sugar (FSS), total non-structural carbohydrate (TNC) minus free simple sugar (FSS) represent starch and non-starch polysaccharide; antifeedant elements include condensed tannin (CT), bioactive tannin (BT) minus condensed tannin (CT) represents hydrolysable tannin, lignin (Ln), hemicellulose (HC), cellulose (Ce).*

## Discussion

### The trade-off in seed phytochemistry of *Lithocarpus*

Our prediction that the seeds inside enclosed receptacle (ER) fruit, with their greater investment in mechanical protection provided by the hard woody receptacle, would contain higher levels of macronutrients and lower levels of antifeedants than the typical acorn fruit, thus representing a trade-off in seed phytochemical defense and investment between the two different fruit types, was generally supported by our results (Table 4). The seeds from enclosed receptacle (ER) fruit possessed higher overall concentrations of macronutrients, especially total non-structural carbohydrate like starch and free simple sugar, and lower overall levels of antifeedants, particularly indigestible fiber. The low concentrations of non-carbohydrate macronutrients, proteins and lipids, observed in all fruit agrees with general patterns observed for other non-commercial nuts species (Vander Wall, 2001), but when the overall pattern of macronutrient investment is taken into account, these important fractions also contribute to the trade-off syndrome (Table 6).

### Fiber is the important antifeedant for *Lithocarpus*

Surprisingly, we found that instead of tannin, indigestible fiber was the most important antifeedants involved in this trade-off strategy (Table 4 and 5). Tannins are considered important antifeedants, as both digestive-inhibitors and toxins [16], [17]. However, in the analysis, we only observed a significantly higher level of condensed tannin (Table 5) in the lowland acorn species (*L. craibianus*) while the relationship was opposed to our predictions in the highlands, where *L. xylocarpus* (ER) had a slightly higher level of bioactive tannin in the seeds than *L. hancei* (acorn). Compared to well-studied tannins, fibers simply act as digestive inhibitors, reducing the efficiency of ingestion and digestion. Our results suggest that the indigestible fiber might be more important and effective in chemical defense for seeds than previously thought and possibly even more important than tannins for these nuts.

The decreased effectiveness of tannin may be due to the taste, salivary glands and digestive system of the foragers [11], [18]. The tannin-binding salivary proteins (TBSPs) are group of proteins in the oval cavity of some rodents that have high affinity for binding tannin and their production is induced by ingesting tannin [17]. This physiological response is considered the first line of defense, as they bind readily to dietary tannins, preventing them from interacting with other proteins. The tannin-binding salivary proteins have been found in the saliva of rats and mice (*Rattus* and *Apodemus*), which were identified as predators and dispersers for *Lithocarpus* seeds [5]. This co-evolutionary response in scatter-hoarding rodents would reduce the effectiveness of tannin as antifeedant, getting the plants into a chemical ‘arms race’. On the other hand, indigestible fibers simply occupy space in the gut and inhibit digestion, but rodents have no physiological mechanism to ameliorate this effect except for passing them quickly through the digestive system. Unfortunately, little is known about the scatter-hoarding rodents that interact with the *Lithocarpus* in this study, so further investigation into the existence of TBSPs in the predators' saliva is necessary to understand the role of tannins.

### Clear trade-off pattern in the lowland sympatric pair

While the general results for sympatric species with contrasting fruit types agree with our trade-off hypothesis, the phytochemical patterns were more clear and consistent in the two lowland species compared to the highland pair (Table 5). In the lowlands, the seeds of *L. craibianus* (acorn) had higher concentrations of all measured antifeedants except cellulose, and the seeds of *L. truncatus* (ER) and lower levels of free simple sugar and total non-structural carbohydrate. As noted above, in contradiction to our hypothesis, *L. xylocarpus* seeds (ER) possessed unusually high level of bioactive tannin while *L. hancei* seeds (acorn) had very low level of crude protein. Rey et al. [19] found a significant interaction in seed predation rates between microhabitat and species at mid-elevations, but not in high-elevation habitats, implying a lower predation risk and higher recruitment rate at high elevation. The greater diversity of seed predators in lowland areas could impose a stronger selection pressure on the chemical evolution of the lowland species, producing a much more clear trade-off pattern compared to the species in the highland. The rodent communities in these tropical and sub-tropical forests in southwestern China are poorly known. Eleven granivore rodent species of the *Niviventer, Rattus, Berylmys, Apodermus, Leopoldamys* and *Micromys* genera comprised the main scatter-hoarders for *L. harlandii* [4], [6]. *Niviventer confucianus* (white-bellied rats) and *N. fulvescens* (chestnuts rats) are two potential scatter-hoarders for *L. glaber* [3]. The difference between our highland and lowland sites in their local rodent communities and the pressures these rodents impose upon the stone oak seed evolution requires further investigation.

### Are ER fruits generally large-seeded?

Seed size is obviously an important characteristic that determines seed fate [9]. Larger seeds experience greater predation than small seeds [20], [21] and they are also more likely to be dispersed a greater distance [22]–[24] and promote seedling recruitment [25]. Because of the ability to recover from significant herbivory damage, larger seeds are also more likely to re-sprout [7]. Moreover, seeds with a harder seed hull had a higher seed removal rate and a reduced instant consumption compare to seeds with softer hulls [26]. Based on previous studies, ER seeds are bigger and better protected by the lignified and thickened husk [14]. While our trade-off hypothesis is supported when the relative concentration levels of macronutrients and antifeedants are considered, the effect of seed size is more complicated. If trade-offs of the investments and defenses are true, given per gram measurements, is it necessarily true that ER fruits should also be larger? This relationship is not so obvious and implies that seed size could evolve in response to other factors. Our results were less conclusive in relation to this question. In the highlands, the highland species (Fig. 3 and 4): ER species (*L. xylocarpus* (ER) is bigger seeded than *L. hancei* (acorn), while the opposite trend was observed for the lowland pair of species, where again the difference might be strongly affected by differences in the community of scatter-hoarders. A more comprehensive study of the situation will be required to understand the effect of seed size on the evolutionary trade-off in seed phytochemistry found in acorn and ER fruit.

### Conclusions

Overall, the two distinctive fruit morphologies of *Lithocarpus*, ER fruits and acorns, exhibit a trade-off strategy in seed phytochemical investment and mechanical protection. Seeds of ER fruits, enclosed by awoody and thickened receptacle, invested more in macronutrients, while seeds of acorns, enclosed by a thin and bitter exocarp, invested more in antifeedants as chemical protection from predation. Carbohydrate and lipid were the most important macronutrient fractions in this trade-off, while surprisingly, acorn seeds demonstrated an increased concentration of indigestible fibers, not tannins, as the important antifeedant participating in this evolutionary pattern. The pair-wise comparison of sympatric ER and acorn species revealed a considerably more clear and consistent trade-off in phytochemistry in the lowlands than the highlands, which may be caused by the different degree of the predation pressure between the two forests.

## Materials and Methods

### *Lithocarpus* and the Study Sites

*Lithocarpus (Blume),* commonly known as stone oaks, range from eastern India through southern China, north to Japan, and extend through Southeast Asia, across the Malayan archipelago to Papua New Guinea [27]. Occupying all elevation zones and most habitats, this genus attains the highest diversity in lower montane forests (1000–2000 m) on relatively poor soils [15]. Approximately 143 *Lithocarpu*s species exist in China, which are restricted to the southern provinces of Yunnan, Guangdong, and Hainan Island [28]. All six samples were collected in Yunnan province, in the Hengduan Mts. Region (Fig. 2). Taxonomic species descriptions of *Lithocarpus* are based primarily upon the fruit morphology. *Lithocarpus* produce hard dry nuts seated in a woody cupule of various forms. The cupule is formed by a much reduced and compressed sterile branching structure [29]. The exact functional nature, if any, of the cupule is poorly understood. Most of the important functional morphology of the fruits relates to the internal structure of the receptacle and acorn, as described above.

We sampled numerous individuals from two pairs of sympatric species with differing fruit morphologies, one each in the lowlands and highlands. In the lowlands, the fruits of *L. craibianus* (acorn fruit type) and *L. truncatus* ER fruit type) were collected at the lowland site, Wushigonglichu in Menglun, Xishuangbanna, with elevation ranging from 540m to 800m. This location is on the northern border of the Southeast Asian tropical zone (Figs. 2 and 3). The fruits of the co-dominant *L. hancei* (acorn fruit type) and *L. xylocarpus* (ER fruit type) were collected at the highland site, in the Ailao Mountains, located in Jingdong Xian (Latitude: 24^°^32′N, Longitude: 101^°^01′E) where elevation ranges from 2200 to 2400m. Compared to the two co-dominant highland species, the lowland species are less common and exist in a much more diverse community, both in terms of the trees and other organisms. In addition, several fruit samples were collected opportunistically, including *L. calolepis* (enclosed receptacle fruit) from lower elevations (1500–1800m) in the temperate broad-leaf forest in Ailao Mountains, and *L. grandifolius* (acorn) on the side of the road from Fugong to Liuku, the Hengduan Mts. region (elevation 1112 m, Latitude: 26^°^78.283′N, Longitude: 98^°^88.941′E).

### Fruit Sample Collection, Seed Storage and Seed Size Calculation

The fruits were collected from a total of six species from late October to the beginning of November in 2008 (see Table 2 for sample sizes). The previous year was a major mast-fruiting year for most *Lithocarpus* species in the Hengduan Mts. (pers. obs.). Fruits collected from one individual tree were stored in the same plastic bag marked with the collection information. Seeds were removed from the fruit the same day. Intact seeds from each individual tree then were placed into separate labeled paper envelopes while the seeds damaged by weevils were discarded. The fresh weight of each seed was measured and recorded. The seeds were then stored in the paper envelopes to promote air circulation and prevent the growth of mold. After returning from the field, all the envelopes were placed in a drying oven at 60^°^C for more than a week, after which the total dry weight of the seeds from each envelop was measured and recorded. Total percent water content of the seeds was calculated for each individual tree by subtracting dry weight from fresh seed weight, divided by the total fresh weight of the seeds. Mean seed size per individual was then calculated by taking the total dry weight and dividing it by the number of seeds used for each individual. Before transporting the samples to the nutrient analysis lab, the seeds were sterilized in an –80^°^C freezer for two days and stored in *Nasco* Whirl-pak bags.

### Nutrient Analysis

The nutrient analysis was performed in the Nutritional Ecology Lab of the Human Evolutionary Biology Department of Harvard University. Antifeedant and macronutrient content of the seeds from each individual tree was analyzed. The following antifeedants and macronutrients were measured directly: condensed tannin, total bioactive tannin assayed by radial diffusion, neutral-detergent fiber, acid-detergent fiber, lignin, crude lipid and crude protein.

Crude protein was determined using the Kjeldahl procedure for total nitrogen and multiplying by 6.25 [30]. Insoluble fiber was determined using the Detergent System of Fiber [31] as modified by Robertson and van Soest [32]. Lipid was measured using a modified method of the Association of Official Analytical Chemists [33]. Free simple sugar was estimated using the modified phenol/sulfuric acid colorimetric assay [34], [35]. Condensed tannin content was measured using the method of Bate-Smith [36] as modified by Porter et al. [37]. The content of bioactive tannin was measured using the radial diffusion method [38]. Dry matter was determined by first partially drying the samples in the field, followed by drying a subsample at 100^°^C in a drying oven, for 8h and then hot weighing to obtain the 100% dry correction coefficient. Total ash was measured by ashing the above subsample at 520^°^C in a muffle furnace, for 8 h and then hot weighing at 100^°^C. Complex carbohydrates (starch and nonstarch polysaccharides or soluble fibers) were estimated by subtraction of different fractions, including:

Hemicellulose = Neutral-detergent fiber–Acid-detergent fiber;
Cellulose = Acid-detergent fiber -Lignin;
Total nonstructural carbohydrate = 100−Neutral-detergent fiber-Crude protein-Lipid-Ash.

### Data Analysis

The data analysis was performed using the R statistics package (R Development Core Team, 2008; http://www.R-project.org). The *t-test*s of macronutrient and antifeedant elements were performed for the sympatric species: 1) *L. craibianus* (acorn) and *L. truncatus* (ER) from Wushigonglichu, Menglun (the lowland) and 2) *L. hancei* (acorn) and *L. xylocarpus* (ER) from the Ailao Mountains (the highland). *F-tests* were performed to test the variance among species. The levels of macronutrients and antifeedants of the sympatric species were standardized into the percentage of the field dry matter for ER and acorn group comparison. *Analysis of variance* (*ANOVA)* and *MANOVA* were performed to compare the antifeedants and macronutrients content of the seeds between two fruit types. *Discriminant Function Analysis* (*DA*) was performed to further test the chemical partitioning difference between the acorn and ER species' seeds. Some chemical elements were reformatted for the *DA* analysis: Starch and non-starch polysaccharides are represented by total nonstructural carbohydrate minus free simple sugar; hydrolysable tannin is represented by bioactive tannin minus condensed tannin. Data standardization was performed before the *DA* analysis.

## References

[1] Vander Wall SB (2001). The evolutionary Ecology of fruit dispersal.. Bot Rev.

[2] Vander Wall SB (2002). Masting in Animal-dispersed pines facilitates seed dispersal.. Ecology.

[3] Zhang T, Li K, Cai Y, Yang K, Hu X (2006). Predation and dispersal of *Lithocarpus glaber* seeds by rodents in Tiantong National Forest Park, Zhejiang Province.. Chin J Appl Ecol.

[4] Xiao Z, Zhang Z, Wang Y (2003). Observations on tree seed selection and caching by Edward's long-tailed rat (*Leopoldamys edwardsi*).. Acta Theriol Sin.

[5] Xiao Z, Wang Y, Harris M, Zhang Z (2006). Spatial and temporal variation of seed predation and removal of sympatric large-seeded species in relation to innate seed traits in a subtropical forest, Southwest China.. For Ecol Manage.

[6] Xiao Z, Zhang Z (2006). Nut predation and dispersal of Harland Tanoak Lithocarpus Harlandii by scatter-hoarding rodents.. Acta Oecol.

[7] Wang B, Chen J (2009). Seed size, more than nutrient or tannin content, affects seed caching behavior of a common genus of Old World rodents.. Ecology.

[8] Van Soest PJ (1994). Nutritional ecology of the ruminant..

[9] Jansen PA, Forget PM (2001). Scatter-hoarding rodents and tree regeneration.. Dynamics and Plant-Animal Interactions in a Neotropical Rainforest.

[10] Wood MD (2005). Tannin and lipid content of acorns in scatterhoards and larderhoards.. Northeast Nat.

[11] Wrangham RW, Conklin NL, Hunt KD (1998). Dietary response of chimpanzees and cercopithecines to seasonal variation in fruit abundance: I. Antifeedants.. Int J Primatol.

[12] Robbins CT (2001). Wildlife feeding and nutrition..

[13] Steele MA, Knowles T, Bridle K, Simms EL (1993). Tannins and partial consumption of acorns: implications for dispersal of oaks by seed predators.. Am Midl Nat.

[14] Cannon CH, Manos PS (2000). The Bornean *Lithocarpus* Bl. section *Synaedrys* (Lindl.) Barnett (Fagaceae): its circumscription and description of a new species.. Bot J Linn Soc.

[15] Cannon CH, Manos PS (2001). Combining and comparing continuous morphometric descriptors with a molecular phylogeny: the case of fruit evolution in the Bornean *Lithocarpus* (Fagaceae).. Syst Biol.

[16] Shimada T (2001). Nutrient compositions of acorns and horse chestnuts in relation to seed-hoarding.. Ecol Res.

[17] Shimada T (2006). Salivary proteins as a defense against dietary tannins.. J Chem Ecol.

[18] Wang B, Chen J (2011). Scatter-hoarding rodents prefer slightly astringent food.. PLoS ONE.

[19] Rey PJ, Garrido JL, Alcantara JM, Auilera A, Garcia L (2002). Spatial variation in ant and rodent post-dispersal predation of vertebrate- dispersed seeds.. Funct Ecol.

[20] Schoener T W (1971). Theory of feeding strategies.. Annu Rev Ecol Syst.

[21] Smallwood PD, Peters WD (1986). Grey squirrel food preferences: the effects of tannin and fat concentration.. Ecology.

[22] Stapanian MA, Smith CC (1978). A model for seed scatterhoarding: Coevolution of fox squirrels and black walnuts.. Ecology.

[23] Clarkson K, Eden SF, Sutherland WJ, Houston AI (1986). Density dependence and magpie food hoarding.. J Anim Ecol.

[24] Jansen PA, Bartholomeus M, Bongers F, Elzinga JA, Denouden J (2002). The role of seed size in dispersal by a scatterhoarding rodent.. Seed dispersal and Fruigivory: Ecology, Evolution and Conservation.

[25] Crawley MJ, Long CR (1995). Alternating bearing, predator satiation and seedling recruitment in *Quercus robber L*. J Ecol.

[26] Jacobs F (1992). The effect of handling time on the decision to cache by grey squirrels.. Anim Behav.

[27] Soepadmo E (1972). Fagaceae..

[28] Chun W, Huang C (1998). Flora of China..

[29] Forman LL (1966). On the evolution of the cupules in the Fagaceae.. Kew Bull.

[30] Pierce WC, Haenisch EL (1947). Quantitative analysis (2^nd^ edn)..

[31] Goering HK, Van Soest PJ (1970). Forage Fiber Analysis..

[32] Robertson JB, Van Soest PJ (1980). The detergent system of analysis and its application to human foods.. The analysis of dietary fiber in foods.

[33] Association of Official Analytical Chemists (AOAC) (1984). Fat (crude) or ether extract in animal feeds: direct method..

[34] Strickland JDH, Parsons TR (1972). A practical handbook of seawater analysis..

[35] Dubios M, Gilles KA, Hamilton JK, Rebers PA, Smith F (1956). Colorimetric methods for determination of sugars and related substances.. Anal Biochem.

[36] Bate-Smith EC (1975). Phytochemistry of proanthocyanidins.. Phytochemistry.

[37] Porter LJ, Hrstich LN, Chan BG (1986). The conversion of procyanidins and prodelphinidins to cyaniding and delphinidin.. Phytochemistry.

[38] Hagerman AE (1987). Radial diffusion method for determining tannin in plant extracts.. J Chem Ecol.

